# The Glutathione Metabolite γ-Glutamyl-Glutamate Partially Activates Glutamate NMDA Receptors in Central Neurons With Higher Efficacy for GluN2B-Containing Receptors

**DOI:** 10.3389/fphar.2021.794680

**Published:** 2022-01-03

**Authors:** Fatiha Sebih, Nawfel Mokrane, Pierre Fontanel, Mete Kayatekin, Mahira Kaabeche, Janique Guiramand, Catherine Cohen-Solal, Thierry Cens, Matthieu Rousset, Pierre Charnet, Marie-Céleste De Jésus Ferreira, Jean-Baptiste Thibaud, Claudine Ménard, Sonia Cantel, Valérie Rolland, Michel Vignes, Julien Roussel

**Affiliations:** UMR5247 IBMM University of Montpellier, Montpellier, France

**Keywords:** NMDA receptors, GluN2B, GSH metabolism, gamma-Glu-Glu, partial effect

## Abstract

Gamma-L-glutamyl-L-glutamate (γ-Glu-Glu) was synthetized and further characterized for its activity on cultured neurons. We observed that γ-Glu-Glu elicited excitatory effects on neurons likely by activating mainly the N-methyl-D-aspartate (NMDA) receptors. These effects were dependent on the integrity of synaptic transmission as they were blocked by tetrodotoxin (TTX). We next evaluated its activity on NMDA receptors by testing it on cells expressing these receptors. We observed that γ-Glu-Glu partially activated NMDA receptors and exhibited better efficacy for NMDA receptors containing the GluN2B subunit. Moreover, at low concentration, γ-Glu-Glu potentiated the responses of glutamate on NMDA receptors. Finally, the endogenous production of γ-Glu-Glu was measured by LC-MS on the extracellular medium of C6 rat astroglioma cells. We found that extracellular γ-Glu-Glu concentration was, to some extent, directly linked to GSH metabolism as γ-Glu-Glu can be a by-product of glutathione (GSH) breakdown after γ-glutamyl transferase action. Therefore, γ-Glu-Glu could exert excitatory effects by activating neuronal NMDA receptors when GSH production is enhanced.

## Introduction

Gamma-glutamyl dipeptides (γ-Glu-AA) are naturally occurring compounds which result from the transfer of an amino acid moiety on the carboxyl group in the gamma position of glutamic acid. In cells, these compounds are possibly yielded by the action of two enzymes: either gamma-glutamyl transferase (γ-GT) or glutamate cysteine ligase (GCL) (Bachhawat and Yadav, 2018). γ-Glu-AA synthesis is therefore linked to glutathione (GSH) metabolism. Indeed, the first step of glutathione (γ-Glu–Cys–Gly) synthesis, that is, the condensation of cysteine and on the gamma carboxylic group of glutamate is catalyzed by GCL. In addition, γ-GT is involved in GSH/GSSG degradation (Lu, 2013). To some extent, γ-Glu-AA production may reflect the activity of the GSH cycle, and thus, indirectly, the cell antioxidant builds up defense against oxidative stress. In this line, changes in seric γ-Glu-AA concentration are considered biomarkers of liver diseases (Kobayashi et al., 2020) and neurodegenerative diseases (Nemes et al., 2001). They could also play a critical role in the regulation of the central concentration of the neurotransmitters glutamate and GABA, as shown when a ketogenic diet is applied to treat epilepsy (Olson et al., 2018).

As these dipeptides contain glutamate in their structure, some of them exhibit high affinity for ionotropic glutamate receptors, including AMPA, kainate, and NMDA receptors (AMPAr, KAr, and ANMDAr), as evidenced recently by binding studies (Tamborini et al., 2016). In fact, the affinity for NMDA receptors appears to be a broad feature shared by many γ-glutamyl–substituted derivatives. Indeed, it is known for long that γ-D-Glu-Gly displays NMDA receptor antagonist activity (Francis et al., 1980). In this line, our laboratory has shown recently that the green tea amino acid L-theanine (or L-γ-N-ethyl-glutamine) potentiated NMDA responses on hippocampal neurons by exhibiting a partial agonist activity of the glycine site of NMDA receptors (Sebih et al., 2017a).

In the present study, we aimed first to develop a new, simple, and efficient synthesis of γ-glutamyl dipeptides and second to characterize their effect on glutamatergic neurotransmission. This step was performed, thanks to calcium imaging and electrophysiological recordings on cultured neuronal cells and on cells expressing glutamate receptors, including C6 astroglioma cells and *Xenopus* oocytes. Third, we aimed to examine the link between the GSH cycle and γ-Glu-AA production. With this aim, the γ-Glu-AA concentration was measured by LC-MS on C6 cell supernatants treated with GSH synthesis modulators.

For this study, we have focused on the characterization of γ-Glu-Glu as its occurrence has been evidenced in the brain (Reichelt, 1970), and as it can be released by depolarization from the hippocampal slices (Li et al., 1996). In addition, it has better affinity for ionotropic glutamate receptors among other γ-Glu-AAs (Tamborini et al., 2016).

## Results

### Chemical Synthesis of γ-Glu-Glu

The gamma-glutamyl dipeptide γ-L-Glu-L-Glu has been prepared using conventional methods for peptide synthesis. The synthesis of gamma-glutamyl dipeptides was described using a flow chemistry reactor in combination with an immobilized coupling reagent (polymer-supported (PS-HOBt) and scavengers (Baxendale et al., 2006). The authors avoided purification and chromatography steps, but they had to perform the immobilization of HOBt and two elutions, respectively, on Amberlyst A21 (to remove HCl) and Amberlyst A-15 for the coupling reaction (Tamborini et al., 2016).

**SCHEME 1:**
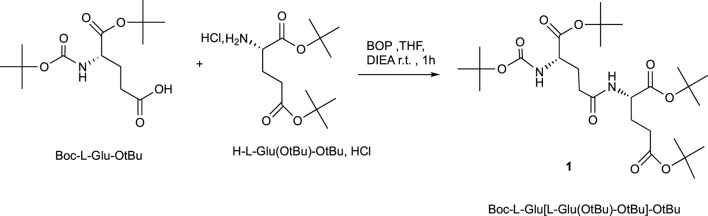
Peptide coupling procedure.

The synthesis of H-Glu (Glu-OH)-OH described in Scheme 1 started with suitably commercial protected amino acid without purification. Boc-L-Glu-O*t*Bu was dissolved in THF in the presence of BOP, Castro’s reagent as the coupling reagent, di-isopropylethylamine (DIEA) as the base, and finally the protected amino acid hydrochloride H-L-Glu (O*t*Bu)-O*t*Bu, HCl was added; the pH of the mixture was adjusted at 8.5 with DIEA for optimum coupling. The mixture was stirred for 1 h at room temperature. After acid–base treatments and purification, the dipeptide Boc-L-Glu [L-Glu (O*t*Bu)-O*t*Bu]-O*t*Bu ***1*** was obtained in a 92% yield.

**SCHEME 2:**
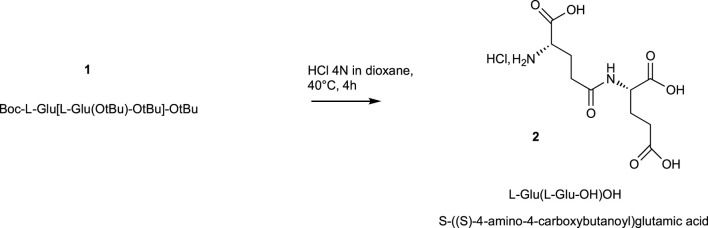
Hydrolysis of protecting groups.

The final compounds γ-L-H-Glu(L-Glu-OH)OH ***2*** ((*S*,*S*) 4-amino-4-carboxybutanoyl) glutamic acid) as chlorhydrate salt were obtained in 90% yield by removing *N*-BOC and *tert*butyl esters protecting groups in acidic conditions using HCl 4N aqueous solution in dioxane (Scheme 2). This step was monitored by analytical HPLC to follow the disappearance of all protecting groups and to check default of racemization. This acidic hydrolytic step can also be performed with 80% trifluoroacetic acid solution in CH_2_Cl_2_ in very high yield. However, trifluoroacetic salts are toxic for the cells.

### Effect of γ-Glu-Glu on Glutamate Receptors in Cultured Hippocampal Cells

The binding studies led by Tamborini *et al* have shown that γ-Glu-Glu has the best binding affinity for all ionotropic glutamate receptors, including AMPA, kainate, and NMDA receptors (AMPAr, KAr, and ANMDAr), compared to other γ-Glu-AAs (Tamborini et al., 2016). But, the pharmacological activities of this dipeptide on these receptors are unknown.

**FIGURE 1:**
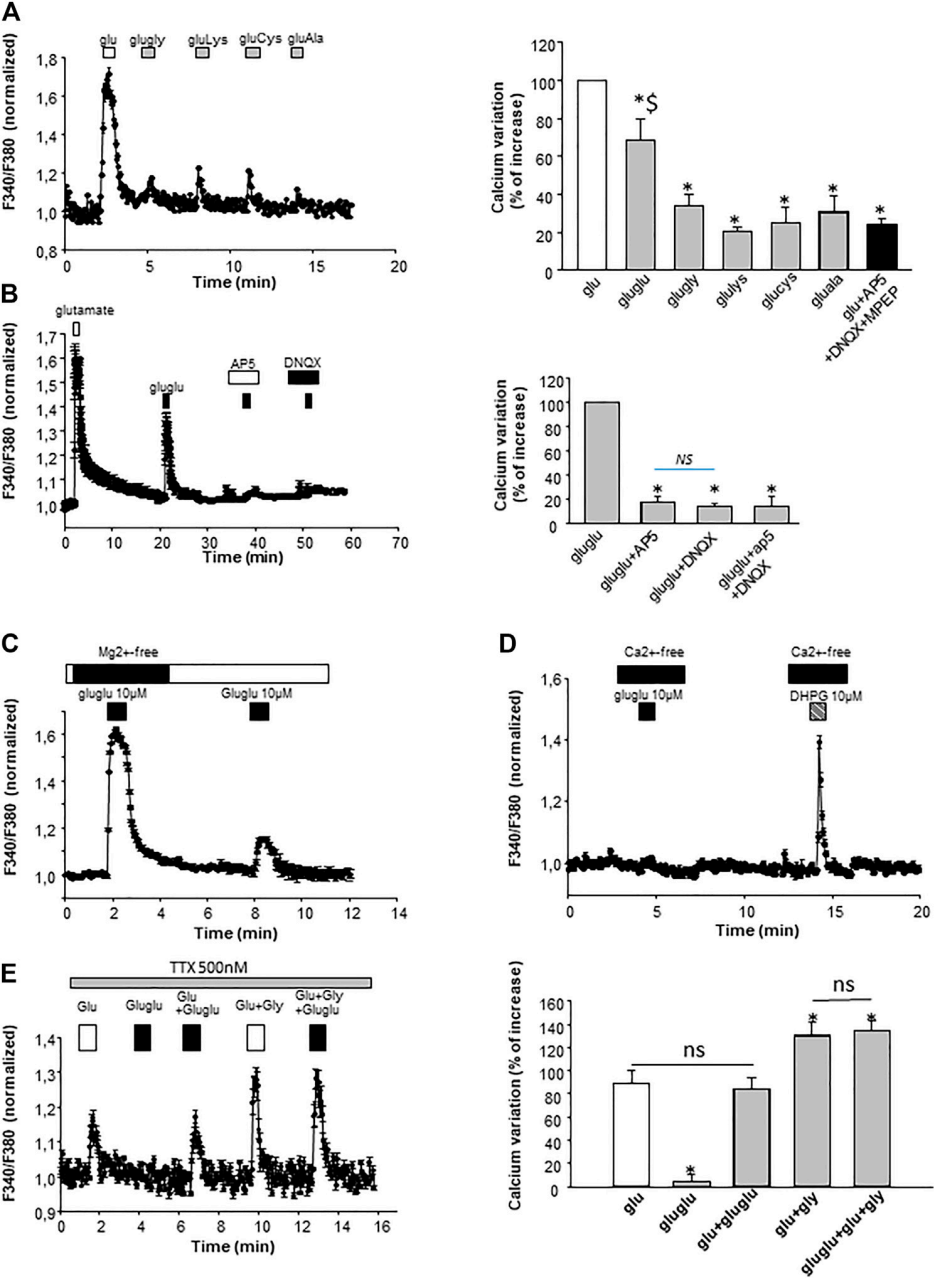
Characterization on the γ-Glu-AA effect on [Ca^2+^]_i_ in cultured neurons. **(A)** γ-Glu–Gly, γ-Glu–Lys, γ-Glu–Cys, and γ-Glu–Ala were sequentially applied at 10 µM on cultured neurons. On the left, an illustrative sample trace obtained by averaging [Ca^2+^]_i_ changes recorded in the three cells is shown. On the right, the recapitulative graph plot peak [Ca^2+^]_i_ normalized to glutamate response (at 10 µM; 100%) for all the γ-Glu-AA tested (*n* = 75). *p < 0.05 when comparing γ-Glu-AA- *vs* the Glu-elicited response; $ *p* < 0.05 when comparing γ-Glu-Glu- *vs* other γ-Glu-AA–elicited responses. **(B)** Pharmacological characterization of γ-Glu-Glu targets with glutamate ionotropic receptor antagonists AP5 (25 μM; NMDAr antagonist) and DNQX (10 μM; AMPAr and KAr antagonist). On the left, a graph plotting averaged sample traces of the 27 cells recorded during one individual experiment is shown. On the right, the recapitulative graph plot peak [Ca^2+^]_i_ normalized to γ-Glu-Glu response (at 10 μM; 100%) for all the conditions tested (*n* = 193). **(C)** Illustrative experiment depicting the dependency of γ-Glu-Glu–elicited [Ca^2+^]_i_ increase on the presence of Mg^2+^ ions in the extracellular medium. The graph was obtained by averaging traces recorded in the 30 individual cells. **(D)** Representative experiment illustrating the dependency of γ-Glu-Glu effects on the extracellular Ca^2+^ ions. The metabotropic glutamate receptor (mGlu5) agonist DHPG (10 µM) was tested as a control for the stimulation Ca^2+^ release from intracellular stores. The graph has been generated by averaging the responses of 25 individual cells recorded. **(E)** Effect of TTX of γ-Glu-Glu–elicited responses. On the left, a graph plotting the responses of Glu and γ-Glu-Glu is shown. It has been obtained by averaging [Ca^2+^]i changes from 12 individual cells recorded during a single experiment. On the right, the graph recapitulates the [Ca^2+^]i increases elicited by Glu, γ-Glu-Glu, Glu+γ-Glu-Glu, γ-Glu-Glu + Gly, and Glu+γ-Glu-Glu + Gly. Data are presented as [Ca^2+^]i increases normalized to the responses of Glu (*n* = 68).

The first objective of this study was to determine if γ-Glu-Glu activated glutamatergic receptors. With this aim, intracellular Ca^2+^ ([Ca^2+^]_i_) changes were monitored in cultured hippocampal neurons in the presence of γ-Glu-AAs. When Mg^2+^ ions were omitted from the extracellular medium [Ca^2+^]_i_ increases were observed in the presence of γ-Glu-Glu (Figure 1A). Its activity was further compared to that elicited by other γ*-*L-Glu-AAs which exhibit good affinity for one or more ionotropic glutamate receptors, that is, γ-L-Glu–Lys, γ-L-Glu–Gly, γ-Glu–Cys, and γ-Glu–Ala. When tested at 10 μM, γ-Glu-Glu was the only one among the *γ-*L-Glu-AAs tested to elicit significant excitatory action evidenced by a Ca^2+^ increase (68.5 ± 11.10% after normalizing the response elicited by the dipeptides to the response induced by 10 µM glutamate; *n* = 75). This response was significantly different from the “background” response which was obtained by applying Glu in the presence of glutamate receptor blockers, namely, AP5 25 μM, a broad-spectrum NMDAr antagonist, DNQX 10 μM, a non-NMDA receptor antagonist, and MPEP 10 μM, a type 5 metabotropic glutamate receptor antagonist.

**FIGURE 2:**
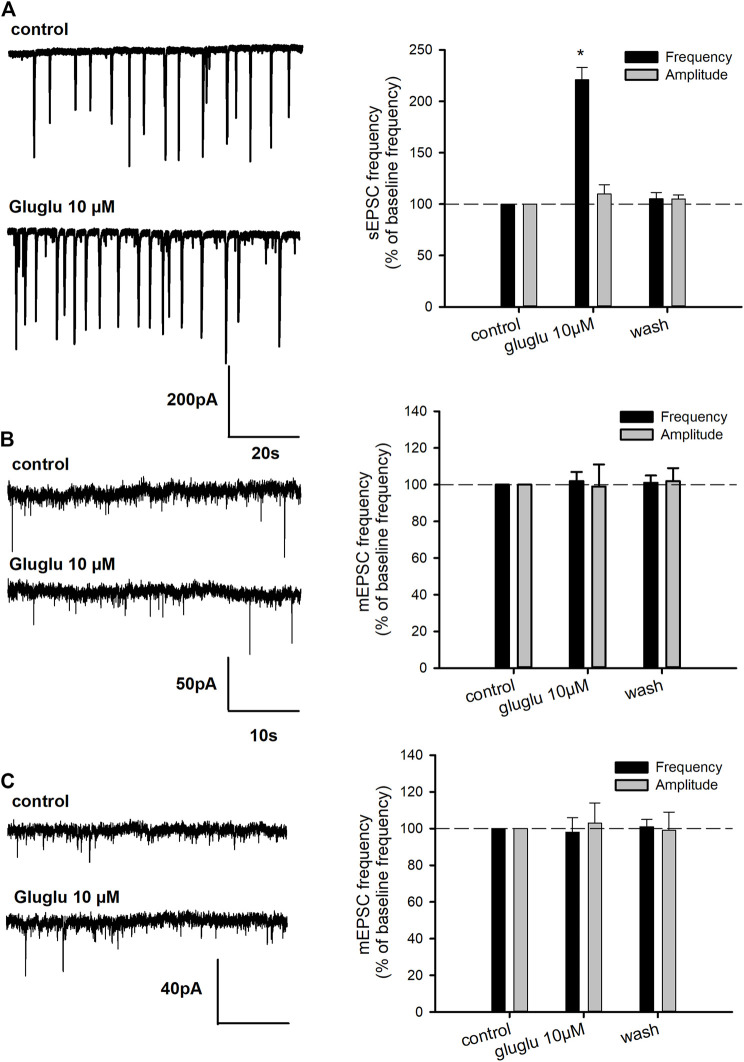
Effect of γ-Glu-Glu on spontaneous excitatory synaptic transmission. **(A)** On the left, representative traces showing the exciting effect of γ-Glu-Glu on sEPSC recorded in the Mg^2+^-free medium. A recapitulative graph plotting the changes in sEPSC frequency and amplitude is shown. Data are expressed as averages (±SEM) of sEPSC frequencies or amplitudes, normalized to respective basal sEPSC frequency or amplitude. *p < 0.05 *vs.* baseline frequency (one-way ANOVA followed by the Holm–Sidak t-test) **(B)** Miniature spontaneous transmission (mEPSC) was recorded in the presence of TTX (500 nM) in a Mg^2+^ ion–containing extracellular medium. On the left, the extracts of mEPSC recordings obtained in the control and in the presence of γ-Glu-Glu are shown. On the right, a graph plot mEPSC frequency and amplitude. Data are expressed as averages (±SEM) of mEPSC frequencies or amplitudes normalized to the respective basal mEPSC frequency or amplitude. **(C)** Miniature EPSCs were recorded in the presence of TTX (500 nM) in a Mg^2+^ ion–free medium. On the right, a graph plot mEPSC frequency and amplitude is shown. Data are expressed as averages (±SEM) of mEPSC frequencies or amplitudes normalized to the respective basal sEPSC frequency or amplitude.

In order to further characterize the excitatory effect of γ-Glu-Glu, it was applied in the presence of NMDAr or AMPAr/KAr antagonists, that is, AP5 or DNQX, respectively. The application of ionotropic glutamatergic receptor inhibitors prevented γ-Glu-Glu–induced Ca^2+^ increase independently of each other. This result indicates that γ-Glu-Glu*–*induced Ca^2+^ increase involved NMDAr and/or AMPAr. The [Ca^2+^]_i_ increases observed in cultured neurons under these conditions may result from a direct excitatory action by binding ionotropic receptors and/or from an indirect effect involving the stimulation of endogenous glutamate synaptic release. In order to test this hypothesis, experiments were carried out in the presence of tetrodotoxin (TTX, 500 nM) in order to block synaptic transmission. In the presence of TTX, γ-Glu-Glu–elicited responses were completely blocked, indicating that global [Ca^2+^]_i_ increases resulted from the excitatory action of γ-Glu-Glu *via* NMDAr activation and subsequent AMPAr and NMDAr activation likely by endogenous glutamate release. As expected, the potentiating effect of glycine on NMDAr-mediated responses was still observed in the presence of TTX. In addition, this response was unchanged in the presence of γ-Glu-Glu, suggesting no direct additive action of glycine and γ-Glu-Glu on glutamate-mediated actions under these conditions. Such an excitatory effect of γ-Glu-Glu on synaptic transmission was further examined by recording spontaneous glutamate-mediated synaptic transmissions. γ-Glu-Glu increased spontaneous excitatory postsynaptic current (sEPSC) frequency. Spontaneous EPSCs were recorded in an Mg^2+^-free state to uncover NMDA receptor–mediated synaptic events (Figure 2A). The application of γ-Glu-Glu significantly and reversibly enhanced the frequency of sEPSC (220 ± 12% of basal frequency; *n* = 3). This excitatory effect was not observed on mEPSCs isolated by applying TTX either in the presence (*n* = 3) or absence (*n* = 4) of Mg^2+^ ions in the extracellular medium (Figures 2B,C, respectively). Both the mean amplitude and frequency of appearance were unaffected by γ-Glu-Glu application. This result precludes that presynaptic NMDA receptors are involved in the excitatory effect of γ-Glu-Glu on synaptic transmission. Taken together, these data confirmed those obtained by measuring intracellular Ca^2+^ changes in the presence of TTX.

### Characterization of γ-Glu-Glu Actions on Cells Expressing NMDAr

Taken together, our data raised on hippocampal neurons suggest that γ-Glu-Glu behaves as a partial agonist of NMDA receptors. In order to further characterize this effect, γ-Glu-Glu was then tested on cells expressing either of GluN1 and GluN2A or GluN1 and GluN2B NMDAr subunits, bearing in mind that they are the most abundant NMDA receptors found in the hippocampus (Shipton and Paulsen, 2014).

**FIGURE 3:**
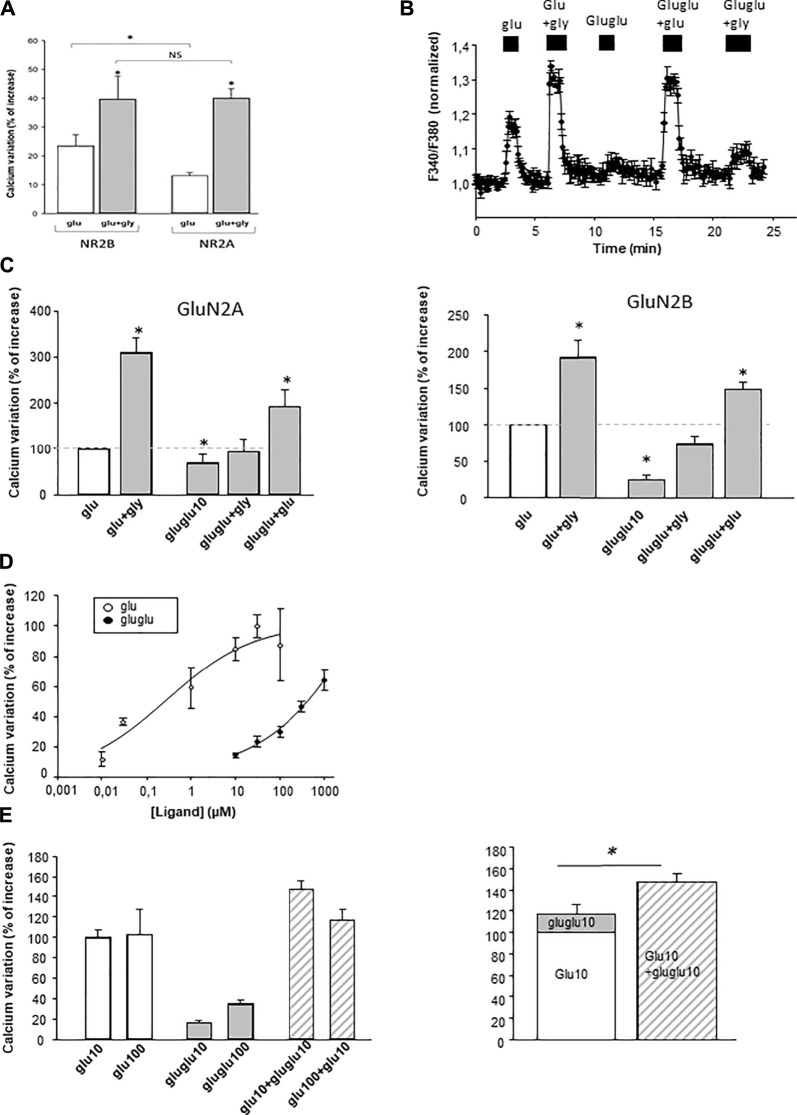
Effect of γ-Glu-Glu on [Ca^2+^]i on C6 cells transfected with the NMDAr. **(A)** Comparative effect of 10 µM glutamate and 10 µM glutamate + glycine application in the NMDAr comprising the GluN2A or Glu2B subunit (*n* = 100). **(B)** Illustrative experiment depicting the effect of γ-Glu-Glu and Glu, both applied at 10 µM in the presence or not of glycine (gly, 10 µM) on the GluN2B-expressing C6 cells (*n* = 3). **(C)** Recapitulative graphs plotting the effects of γ-Glu-Glu in the presence of Glu or Gly on GluN2A- (left; *n* = 10) and GluN2B-expressing cells (right; *n* = 17). **(D)** Concentration-dependent curve of the effect of Glu and γ-Glu-Glu on [Ca^2+^]_i_ measured in GluN2B-expressing cells (*n* = 15 each). **(E)** Quantitative analysis of the effect of the combination of Glu (10 µM or 100 µM) with γ-Glu-Glu (10 µM) (left, *n* = 15). On the right, the graph compares the theoretical response obtained by adding the individual responses elicited by Glu (10 µM) and of γ-Glu-Glu (10 µM) (right histogram) with the experimental response obtained by co-applying Glu (10 µM) with γ-Glu-Glu (10 µM). *p < 0.05 when comparing both conditions (one-way ANOVA followed by the Holm–Sidak t-test).

NMDA receptors were expressed first in astroglioma C6 cells. We have first checked that functional NMDArs were correctly expressed in this cell line. First, glutamate elicited a [Ca^2+^]_i_ rise only in transfected cells. Second, this response was potentiated by glycine (10 µM), inhibited NMDAr antagonist AP5, and in the presence of Mg^2+^ ions in the extracellular medium (Figure 3A). The transfected NMDArs thus retained their functional properties when transfected in C6 cells. We also observed that GluN2B-expressing cells were more sensitive to glutamate than GluN2A-expressing cells. Gamma-Glu-Glu–elicited [Ca^2+^]_i_ increases were detected when it was applied at a concentration of 10 µM on both GluN2A- and GluN2B-expressing C6 cells (Figures 3B,C). It had no effect on untransfected cells (data not shown). In addition, glycine potentiated γ-Glu-Glu responses. Gamma-Glu-Glu dose-dependently increased [Ca^2+^]_i_ with an EC50 value circa 300 µM. It was less potent than glutamate which exhibited an EC50 value in the micromolar range (Figure 3D). Therefore, γ-Glu-Glu displayed partial agonist effect on NMDA receptors. In this line, γ-Glu-Glu was further tested in combination with Glu to evaluate whether it could antagonize Glu effects. In fact, we observed that γ-Glu-Glu when tested at 10 µM consistently potentiated the responses elicited by Glu at 10 µM. However, this potentiation was lost when Glu was tested at 100 µM (Figure 3E). Therefore, γ-Glu-Glu does not really behave as a “conventional” partial agonist as it does not elicit any blocking action on glutamate-evoked responses.

**FIGURE 4:**
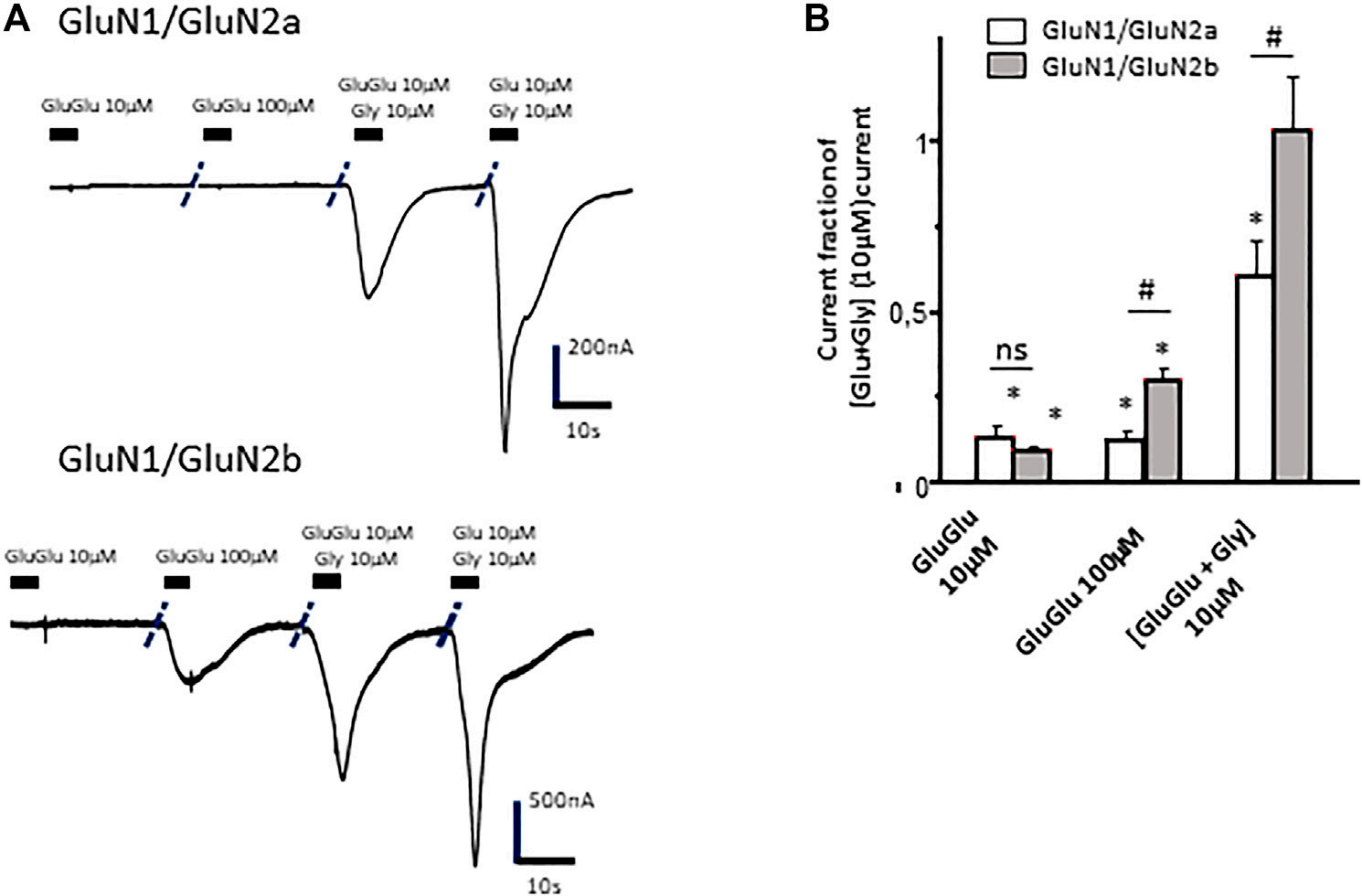
Effect of γ-Glu-Glu on GluN1/GluN2A- and GluN1/GluN2B NMDAr-expressing *Xenopus* oocytes. **(A)** Current traces obtained from GluN1/GluN2A- **(top)** and GluN1/GluN2B- **(bottom)** expressing oocytes in the presence of γ-Glu-Glu and Glu alone or in combination with glycine. **(B)** Recapitulative graph plotting current amplitudes expressed as fractions of the current obtained by co-applying Glu and Gly (each at 10 µM) in both GluN1/GluN2A- and GluN1/GluN2B-expressing oocytes. Data are presented as averages (±SEM) of 12 independent experiments. * indicates a significant difference between a given condition and the glutamate + glycine effect. ^#^ indicates a significant difference between the current amplitudes recorded on GluN2A-expressing oocytes and those recorded on GluN2B-expressing oocytes (two-way ANOVA).

Concentration-dependent effects of γ-Glu-Glu were further evaluated on oocytes expressing GluN2A- or GluN2B-containing NMDA receptors. Interestingly, γ-Glu-Glu elicited different responses according to the concentration applied and to NMDAr subunit composition. GluN2A-containing NMDArs were more sensitive to low concentrations of γ-Glu-Glu than GluN2B-containing NMDArs. At higher concentrations (100 µM), the opposite was observed. Such a discrepancy was retained when γ-Glu-Glu was applied in the presence of glycine (Figures 4A,B). This was also observed on C6 cells expressing NMDAr.

### γ-Glu-Glu Production by Astroglioma Cells Depends on Glutathione Metabolism

**FIGURE 5:**
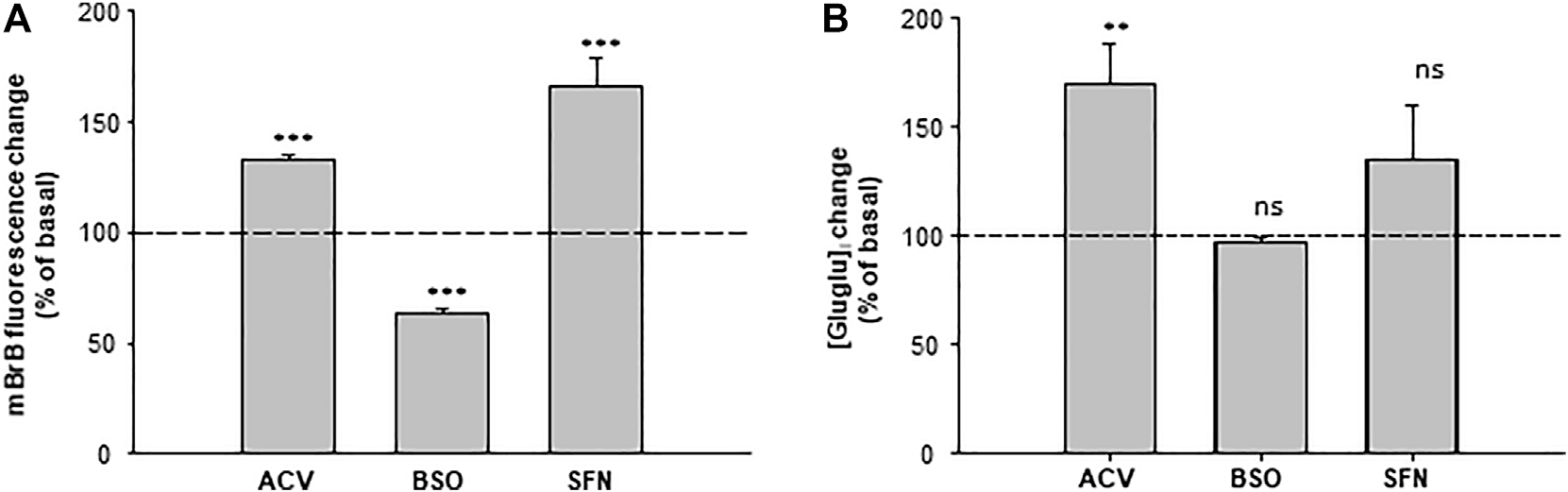
Quantification of γ-Glu-Glu in the extracellular medium of astroglioma C6 cells. **(A)** [GSH]_i_ content in the control, BSO-, ACV-, and SFN- pretreated C6 cells. GSH was measured *in cellulo* with monobromobimane (mBrB) 24 h following the treatments. On the graph, data are presented as percentages of mBrB fluorescence intensity normalized to basal mBrB fluorescence measured in the control-untreated cells. ***p < 0.001 when comparing mBrB fluorescence measured in the control cells with either treatment (one-way ANOVA followed by the Holm–Sidak t-test). **(B)** Measurement of [γ-Glu-Glu] in the extracellular medium of C6 cells. Analysis of the C6 extracellular medium was performed on 100 µl samples of the culture medium of cells treated with BSO, ACV, and SFN. On the graph, [γ-Glu-Glu]e has been normalized to [γ-Glu-Glu]e detected in the control-untreated cells. **p < 0.01 and “not significant” (ns) when comparing [γ-Glu-Glu]e measured in control cells with either treatment (one-way ANOVA followed by the Holm–Sidak t-test).

In the brain, γ-Glu-AAs can be obtained after GSH lysis by γ-GT. They are further released in the extracellular space. In addition, astrocytes appear to be the major source of γ-Glu-AAs (Dringen et al., 1999). We have thus evaluated whether γ-Glu-Glu was found in the extracellular medium of cultured C6 astroglioma cells and whether its concentration was linked to GSH metabolism (Figure 5). For this, the cells were treated either with acivicin (ACV), a blocker of γ-GT, or with L-buthionine-(S, R)-sulfoximine (BSO), a blocker of GCLC and sulforaphane (SFN), which boosts GSH formation by stimulating GCLC expression *via* Nrf2 pathway activation. First, intracellular GSH changes were evaluated in C6 cells in order to confirm the activity of these modulators. BSO (10 µM) significantly reduced GSH as measured *in cellulo* with monobromobimane (mBrB) fluorescence (63 ± 2% of basal; *n* = 3), while ACV (100 µM) and SFN (10 µM) elicited a significant increase in mBrB fluorescence (132 ± 2% and 165 ± 12% of basal, respectively; *n* = 3). The extracellular γ-Glu-Glu concentration ([γ-Glu-Glu]_e_) was further measured, thanks to LC-MS. Basal [γ-Glu-Glu]_e_ was 262 ± 56 nM (*n* = 3). In the presence of ACV, [γ-Glu-Glu]_e_ was significantly increased to 170 ± 17% of the basal concentration (*n* = 3). BSO had merely no effect on [γ-Glu-Glu]_e_, while SFN enhanced it, although not significantly (135 ± 25%, *n* = 3). Therefore, [γ-Glu-Glu]_e_ tends to be linked to GSH metabolism in astroglioma cells. It is noteworthy that γ-Glu-Glu was not detected in the extracellular medium of cultured neurons (not shown).

## Discussion

We found here that γ-Glu-Glu produced excitatory actions on cultured hippocampal neurons by activating partially NMDA receptors, although γ-Glu-Glu has good affinity for all ionotropic glutamate receptors (Tamborini et al., 2016). This result is in good agreement with a previous study showing such an activity in olfactory bulb neurons (Li et al., 1993). In addition, we also observed that γ-Glu-Glu effects were consistently detected above 10 µM. Nevertheless, γ-Glu-Glu excitatory actions could also involve a positive modulation of glutamate effects. Indeed, at specific concentrations, γ-Glu-Glu potentiated glutamate-elicited responses, while an inhibitory action was more likely expected if it had behaved as a partial agonist of the glutamate binding site on the NMDAr. This suggests that the γ-Glu-Glu binding site could impinge on a positive allosteric and/or co-agonist binding site on NMDAr. Further structural analysis is required to define the potential binding site(s) of this dipeptide on NMDAr. Moreover, we found that γ-Glu-Glu elicited greater selectivity for the NMDAr containing the GluN2B subunit than for those containing the GluN2A subunit. The endogenous occurrence of γ-Glu-Glu could thus favor neurophysiological mechanisms involving GluN2B-containing NMDAr (Sun et al., 2018).

Nevertheless, γ-Glu-Glu elicited excitatory actions at low concentration (10 µM) which were dependent on the integrity of synaptic transmission as TTX (500 nM) blocked both γ-Glu-Glu–elicited [Ca^2+^]i increases and increase in spontaneous synaptic transmission. It is noticeable that γ-Glu-Glu excitatory action was prominently detected on sEPSC frequency than on their amplitude. This most likely arises from the fact that sEPSCs were recorded in Mg^2+^ ion–free medium. Under these conditions, sEPSCs occur as chronic bursts with a steady amplitude and frequency (so-called “network activity”). Moreover, excitations or inhibitions of this activity are detected mainly by changes in the bursting frequency. This is probably due to the fact that network activity results from the synchronized activation of all excitatory synapses at the same time, and as a consequence, modulations are detected mainly by changes in the frequency of burst occurrence. The fact that the excitatory effect of γ-Glu-Glu on synaptic transmission was blocked by TTX indicates that it most likely resulted from an indirect mechanism involving first the partial effect on NMDAr leading and second the endogenous synaptic release of glutamate and glycine, in turn activating both AMPAr and NMDAr (Vignes, 2001). We have previously reported a similar effect with theanine which exerted excitatory effects sensitive to TTX in cultured hippocampal neurons; the excitatory effect of theanine involves a positive modulation of NMDAr (Sebih et al., 2017). The endogenous presence of γ-Glu-Glu could thus be at the origin of the tonic activation of NMDAr which has been identified in the hippocampus (Le Meur et al., 2007). Indeed, Le Meur et al. have shown that NMDArs were chronically activated by ambient glutamate of non-synaptic origin. Thus, γ-Glu-Glu could be involved in this tonic activation of NMDAr since, in addition, it has mainly a glial origin (Dringen et al., 1999). Indeed, gamma-glutamyl dipeptides (γ-Glu-AA) are by-products of glutathione (GSH) metabolism as they result from the breakdown of GSH by the action of γ-GT. They are further released in the extracellular space. We have thus verified whether γ-Glu-Glu production was related to GSH metabolism in astroglial cells. In fact, while boosting GSH cell content with the Nrf2 activator, it resulted, as expected, in an increased occurrence of γ-Glu-Glu. By contrast, rather surprisingly, γ-Glu-Glu concentration was enhanced after treating cells with acivicin, an efficient blocker of γ-GT activity, while a decrease was expected. Nevertheless, as acivicin also enhanced GSH cellular content, one may speculate that there should be another enzymatic route leading to γ-Glu-Glu from GSH. In support of this, Bachhawat and Yadav (2018) indicate that there are alternative pathways to γ-GT activity, including the glutamate–cysteine ligase activity, which could lead to the production of γ-Glu-AA.

The changes in γ-Glu-Glu in the extracellular medium may thus reflect the production of GSH by astroglial cells. As GSH is the major antioxidant in the CNS, increased production of GSH is required in order to build up antioxidant defense during oxidative stress. Therefore, enhanced production of GSH may result in enhanced γ-Glu-Glu, which, in turn, could produce a stronger activation of NMDA receptors. Such an effect could aggravate indirectly the detrimental action of oxidative stress on neurons, which is particularly enhanced in the progression of neurodegenerative diseases.

## Materials and Methods

### 1-Chemical Syntheses

The melting points were obtained using a Büchi 510 capillary apparatus and were uncorrected. ^1^H NMR and ^13^C NMR spectra were recorded at 300 and 75 MHz using a Brüker AC300 instrument and at 600 MHz using a Brüker AC600 instrument. Chemical shifts are quoted in parts per million (ppm) and were referenced to the residual solvent peak. The following abbreviations are used: s, singlet; d, doublet; t, triplet; q, quartet; and m, multiplet. Coupling constants are reported in hertz (Hz). High-resolution mass spectra (HRMS) were recorded on a Micromass Q-TOF electrospray instrument with only molecular ion and other major peaks being reported. LC-MS identification was carried out by electrospray on HPLC Waters Alliance 2690. Flash chromatography was carried out using E-Merck silica gel (Kieselgel 60, 230−400 mesh) as the stationary phase. Thin-layer chromatography (TLC) was carried out on aluminum plates precoated with Merck silica gel 60F254 and visualized by quenching of ultraviolet fluorescence or by staining with a 10% methanol phosphomolybdic acid solution followed by heating. Column chromatography on silica gel was carried out with Merck Kieselgel 60 silica (230–400 mesh). Analytic HPLC was performed on a Waters apparatus 717 plus autosampler with Millenium^32^ program on SymmetryShield^TM^ RP_18_ 3.5 μm 2.1 mm × 20 mm column using a linear gradient of ACN in H_2_O with 0.1% TFA in 5 min with 3 ml/min flow. THF was distilled from sodium/benzophenone ketyl. The reagents were supplied by commercial sources (Merck, Fluka, Sigma-Aldrich, BACHEM, Acros, and Novabiochem).

### General Procedure for Peptide Coupling

#### Synthesis of (S,S) Di-Tert-Butyl (5-(Tert-Butoxy)-4-((Tert-Butoxycarbonyl)Amino)-5-Oxopentanoyl)Glutamate 1

(*S*)Boc-Glu-O*t*Bu (1 mmol, 303.4 mg), (*S*)HCl,H-Glu(O*t*Bu)-O*t*Bu (1 mmol, 295.8 mg), BOP (1.2 mmol, 530.7 mg), and DIEA (3 mmol, 0.52 ml, pH = 8.5) were dissolved in THF (15 ml) and stirred at room temperature. After stirring 1 h, the solvent was removed under reduced pressure, the organic layer was extracted with ethyl acetate (15 ml), and then washed with 2 × 5 ml of 10% citric acid, 2 × 5 ml NaHCO3 (1M), and then with 2 × 5 ml brine. The organic phase was dried over anhydrous MgSO_4_ and, after evaporation of the solvent under reduced pressure, the totally protected dipeptide *1* was purified on a silica gel column in AcOEt/cyclohexane, 7:3, as the eluent.

Yield: 95%; white solid; Rf = 0.58 (AcOEt/cyclohexane, 7:3); tR = 1.98 min. MS (ES+) m/z 544.1.1H NMR (300 MHz, MeOD): δH (ppm) 1.39 (s, 9H, BOC), 1.48–1.49 (m, 27H, 3xOtBu), 2.05 (t, 2H), 2.18 (m, 2H), 2.29 (m, 2H), 2.35 (t, 2H), 3.90 (dd, 1H,CHα, 3J = 9.4 Hz, 3J = 4.27 Hz), 4.27 (dd, 1H,CHα, 3J = 8.95 Hz, 3J = 5.29 Hz).13C NMR (75 MHz, MeOD):δC (ppm) 26.48, 26.6, 27.1, 28.7, 30.43, 32.8, 52.97, 54.22, 79.87, 82.11, 156.58, 171.75, 173.1, 174.13.

#### (S)-2-((S)-4-Amino-4-Carboxybutanoyl) Glutamic Acid 2: γ-L-H-Glu (L-Glu-OH)-OH

The final compound γ-L-Glu (L-Glu-OH)-OH ***2*** was obtained by removing N- and C- protecting groups (i.e., BOC and *tert*butyl esters) in acidic conditions. Boc-L-Glu[L-Glu(O*t*Bu)-O*t*Bu]-O*t*Bu ***1*** (1 mmole, 544 mg) was stirred in HCl 4N aqueous solution in dioxan (10 ml) for 4 h at 40°C. The dioxan was evaporated under reduced pressure, and the N-chlorhydrate salts of gamma dipeptide were precipitated and dried in diethylether. γ-L-Glu (L-Glu-OH)-OH ***2*** was obtained in a 90% yield. It can also be lyophilized.

Yield 90%; hygroscopic white solid; Rf = 0.15 (AcOEt/cyclohexane, 7:3); tR = 0.4 min mp: 98–102°C; Mw C10H26O7N2. MS (ES+) m/z 277.2 (M + H)+. HRMS (ESI) m/z Calcd for [M + H]+ 277.1036, found 277.1041.1H NMR (300 MHz, D2O): δH (ppm) 1.99–2.08 (m, 4H), 2.30–2.41 (m, 2H), 2.39–2.47 (m, 2H), 3.67–3.73 (t, 2H, 2xCHα). 13C NMR (75 MHz, D2O):δC (ppm) 25.55, 26.08, 30.14, 30.74, 53.88, 53.98, 173.80, 173.86, 177.22, 177.56.

### 2-Cell Cultures

Functional characterization of gamma-Glu-Glu was performed on primary hippocampal neurons and cells expressing NMDAr, including transfected astroglioma C6 cells and *Xenopus* oocytes. All experiments were carried out in accordance with the European Community Council Directive of 24th November 1986 (86/609/ECC). Sprague−Dawley rats were obtained from Janvier Laboratories (France). Culture media (DMEM/Ham F12 with HEPES and 4.5 g/L glucose), Dulbecco’s phosphate-buffered saline (Dulbecco’s PBS), Versene, antibiotics, and fetal calf serum (FCS) were purchased from Invitrogen. Culture dishes were obtained from Nunc. All chemicals were obtained from Sigma.

### Hippocampal Neuron-Enriched Cultures

Primary neuronal cultures were established from 18-day-old embryonic rat hippocampi, as previously described (De Jesus Ferreira et al., 2005), with minor modifications. After preincubation with Versene, hippocampal cells were mechanically dissociated and plated at a density of 2 × 106 cells/dish, on 8-well plates containing square (10 × 10 mm^2^) glass coverslips, previously coated with poly(D-lysine) (50 μg/ml) and then with DMEM/HAM F12 containing 10% FCS. The cells were grown in a defined medium containing DMEM/HAM F12, supplemented with 33 mM glucose, 2 mM glutamine, 100 U/ml penicillin, 100 μg/ml streptomycin, 13 mM sodium bicarbonate, 5.5 μg/ml transferrin, 10 μg/ml insulin, 1 pM β-estradiol, 3 nM triiodothyronine, 20 nM progesterone, 5 ng/ml sodium selenite, and 100 μM putrescine. The experiments were performed on cell cultures grown from 6 to 16 DIV.

### C6 Astroglioma Cells

Rat C6 glioma cells were gifted by Dr Nathalie Chevalier (INSERM U1198). They were maintained in DMEM supplemented by 10% fetal calf serum in the presence of 100 U/ml penicillin and 100 μg/ml streptomycin. C6 cells were transfected at 80% confluence with a combination of GluN1 and GluN2A or GluN2B (3.75 µg cDNA/wells) + 10 µl of Lipofectamine (Invitrogen) diluted in Opti-MEM^TM^ (Gibco^TM^). The cells were used 24 h after transfection. All constructions of NMDA receptor subunits have been kindly provided by Pierre Paoletti (IBENS, ENS, Paris). C6 cells were used for less than 15 passages.

### *Xenopus* Oocyte Injection and Oocyte Current Recording

*Xenopus* oocytes were prepared and injected with *in vitro–*transcribed RNA at 1 μg/μl (20−40 nl) of rat GluN1-1a (named GluN1 herein) and rat GluN2A or mouse ε2 (named GluN2B herein) with a stoichiometric ratio of 1:1 (Sebih et al., 2017a). Macroscopic currents were recorded under a two-electrode voltage clamp using a GeneClamp 500 amplifier (Axon Instruments) and analyzed in the ND96 HERG recording solution (in mM, 96 NaCl, 3 KCl, 0.5 CaCl2, 5 HEPES, pH = 7.2). Current and voltage electrodes (less than 1 MΩ) were filled with 3 M KCl. The currents were filtered (20 Hz) and digitized (66 Hz) using a Digidata-1200 interface (Axon Instruments). Data acquisition was performed using version 7 of pClamp software (Axon Instruments). On the graphs depicting pooled data, the currents recorded on oocytes were expressed as fractions of the maximal current obtained on NMDA receptors—expression obtained by applying a combination of glutamate and glycine, both at 10 µM (Yuan et al., 2009).

### 3-Measurement of GSH Content

Changes in the cellular content of GSH were measured after monobromobimane (mBrB) labeling as previously described (De Jesus Ferreira et al., 2005) (Baxter et al., 2015). For this, C6 rat glioma cells were seeded in 96-well culture plates. When reaching 80% confluence, the cells were treated with BSO (10 µM) or SFN (10 µM) or ACV (100 µM) for 24 h. The culture medium was then replaced by the extracellular medium containing 50 µM mBrB. The extracellular medium comprised 124 mM NaCl, 3.5 mM KCl, 25 mM NaHCO_3_, 1.25 mM NaH_2_PO_4_, 1 mM CaCl_2_, 2 mM MgSO_4_, 10 mM D-glucose, and 10 mM HEPES (pH: 7.4). Incubation with mBrB lasted for 30 min, and then the cells were washed with the extracellular medium to remove unbound mBrB. Fluorescence was then measured *in cellulo* with a plate reader (Tecan “Spark” 20M) at 527 nm after excitation at 380 nm. Background fluorescence was obtained from mBrB unlabeled cells. For data processing, the background fluorescence was subtracted to all fluorimetric signals which were further normalized to mBrB fluorescence recorded in control cells. An experimental determination was performed eight times per experiment. The data are expressed as percentages of at least three distinct experiments performed on different cell cultures.

### 4-Measurement of Intracellular Ca^2+^ Concentration

Intracellular calcium concentration ([Ca^2+^]_i_) was measured using the fluorescent indicator fura-2. For this purpose, the cells grown on glass coverslips were loaded with fura-2 by a 30-min incubation at 37°C with 1 μM fura-2-AM and 0.02% Pluronic in the extracellular solution described above. [Ca^2+^]_i_ was monitored by video microscopy. After rinsing, the glass coverslip was transferred to the recording chamber mounted on an inverted microscope (Leica, DMIRB) and continuously superfused with the extracellular medium described above without adding MgSO4 (Mg^2+^ ions free medium). Fura-2 emission was obtained by exciting alternatively at 340 and 380 nm with a rotating filter wheel (Sutter Instruments) and by monitoring emissions (F340 and F380) at 510 nm. Fluorescent signals were collected with a CCD camera (Hamamatsu), digitized, and analyzed with image analysis software (Acquacosmos, Hamamatsu). The ratio of emissions at 510 nm (F340/F380) was recorded in the cells every second. Experiments were carried out at room temperature. Drugs were applied for 1 min with a gravity-fed system. The data are expressed as averages (±SEM) of the ratio between the fura-2 fluorescence values of 340/380 nm excitation wavelength ratios (F340/F380), normalized to the corresponding basal F340/F380 measured prior to any drug application.

Graphs presenting time courses of F340/F380 ratio changes have been obtained by averaging data from a population of cells recorded individually during one single representative experiment. In the graphs of pooled data, “n” values represent the entire population of cells recorded from at least three independent cultures.

### 5-Electrophysiology

Spontaneous excitatory and inhibitory postsynaptic currents (sESPC and sIPSC) were recorded using the whole-cell patch-clamp method. Neurons grown on glass coverslips were transferred to a recording chamber of an upright microscope and continuously superfused with the extracellular medium (in the presence or absence of MgSO4, according to the experimental condition). Experiments were performed at room temperature with glass pipettes (4-5MΩ resistance) filled with the intracellular solution comprising 140 mM CsMeSO_3_, 4 mM NaCl, 1 mM MgCl_2_, 1 mM EGTA, 5 mM HEPES, 2 mM MgATP, and 0.6 mM NaGTP, pH = 7.4 (CsOH). Tetrodotoxin (500 nM) was included to record miniature EPSCs (mEPSCs) (Vignes, 2001). Access resistance was monitored by applying a 10-mV voltage steps. The currents were collected and amplified with an Axoclamp 200B amplifier (Molecular Devices) and digitized (Digidata 1322, Molecular Devices). Spontaneous PSCs were analyzed with John Dempter’s software packages WinEDR and WinWCP. Drugs were applied at the desired concentration *via* a gravity-fed application system.

## 6-Liquid Chromatographic and MS/MS Conditions for γ-Glu-Glu

The extracellular concentration of γ-Glu-Glu was measured with the extracellular medium of cultured rat C6 astroglioma cells. With this aim, C6 cells were grown in 24-well cell culture plates until 80% cell confluence was reached. The cells were further treated with ACV (100 µM) or BS0 (10 µM) or SFN (10 µM). After a 24-h treatment, the samples of 100 µL of the extracellular medium were taken from 500 µL of the total culture well volume. Sample analyses were further conducted using a LC-MS/MS-8050 (Shimadzu Scientific, Inc., Columbia, MD, USA) Triple Quad mass spectrometer (QqQ) equipped with a UHPLC NexeraX2 system.

**FIGURE 6:**
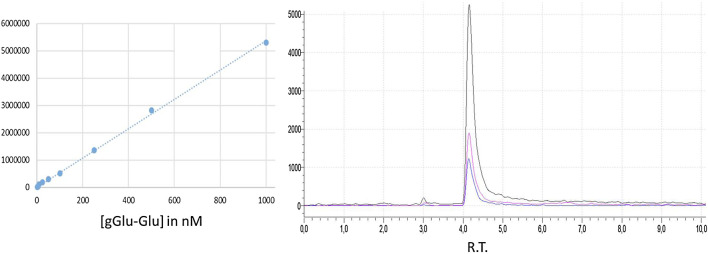
Standard curve for γ-Glu-Glu. γ-Glu-Glu LC-MS/MS in the MRM mode showing the three transitions.

Peak resolution and separation for all samples were optimized by using a Nucleoshell HILIC 50 × 2.1 mm; 2.7 µm (Macherey–Nagel) maintained at 30°C. Mobile phase A: ammonium acetate 100 mM and mobile phase B: ACN/ammonium acetate 100 mM (95/5, v/v) with a 1 ml/min flow and a gradient of 0–80% in 10 min (Figure 6).

System control, data acquisition, and quantitative analysis were performed using Labsolutions software (Shimadzu). Optimal detection conditions were determined from the injection of 594 standard samples over six different parameters. Mass spectrometric detection was operated in positive electrospray ionization and multiple reaction monitoring. The multiple reaction monitoring (MRM) transitions and compound dependent parameters are shown in Table 1. Optimized parameters were obtained by the product ion scan mode of the individual analyte at 10 uM. The parameters for multiple reaction monitoring (MRM) detection in the positive mode are as follows: nebulizing gas flow: 3.0 L/min; heating gas flow: 15 L/min; drying gas flow: 5 L/min; interface temperature: 350°C; desolvation line temperature: 250°C; and heat block temperature: 400°C.

**TABLE 1:** Mass spectrometric parameters: MRM parameters, chromatographic attributes, and quantitative response of γ-Glu-Glu compounds in standard samples.

Analyte	Formula	Mass (Da)	Precursor ion (m/z)	MRM transition m/z	
γ-Glu-Glu	C_10_H_17_N_2_O_7_	276.1	277.1	148.1, 130.0, 84.0 (quanti)	
LOD (mol/L)	LOQ (mol/L)	SD	R^2^	Linearity	Retention time (min)
10-9	10-9	8%	0.999	1000	4.2

Extracellular sample analysis was performed in triplicate per experiment. On the graphs, “n” applies to individual experiments.

### 7-Statistical Analyses

On the recapitulative graphs, “n” applies to the total number of cells recorded and is the sum of all individual cells from at least three independent cell cultures. Statistical analyses were performed using SigmaStat software (Systat software Inc.). One-way ANOVA was generally used for comparison followed by an adequate t-test.

## Data Availability

The raw data supporting the conclusion of this article will be made available by the authors, without undue reservation.
